# Spatial and Temporal Analysis on the Distribution of Active Radio-Frequency Identification (RFID) Tracking Accuracy with the Kriging Method

**DOI:** 10.3390/s141120451

**Published:** 2014-10-29

**Authors:** Xin Liu, Jeremy Shannon, Howard Voun, Martijn Truijens, Hung-Lin Chi, Xiangyu Wang

**Affiliations:** 1 Australasian Joint Research Centre for Building Information Modelling, Curtin University, Bentley WA 6102, Australia; E-Mails: hung-lin.chi@curtin.edu.au (H.-L.C.); xiangyu.wang@curtin.edu.au (X.W.); 2 Woodside Energy Ltd., Perth 6000, Australia; E-Mails: jeremy.shannon@woodside.com.au (J.S.); howard.voun@woodside.com.au (H.V.); martijn.truijens@woodside.com.au (M.T.); 3 Department of Housing and Interior Design, Kyung Hee University, Seoul 130-171, Korea

**Keywords:** radio frequency identification (RFID), spatial analysis, spatial and temporal variation, tracking

## Abstract

Radio frequency identification (RFID) technology has already been applied in a number of areas to facilitate the tracking process. However, the insufficient tracking accuracy of RFID is one of the problems that impedes its wider application. Previous studies focus on examining the accuracy of discrete points RFID, thereby leaving the tracking accuracy of the areas between the observed points unpredictable. In this study, spatial and temporal analysis is applied to interpolate the continuous distribution of RFID tracking accuracy based on the Kriging method. An implementation trial has been conducted in the loading and docking area in front of a warehouse to validate this approach. The results show that the weak signal area can be easily identified by the approach developed in the study. The optimum distance between two RFID readers and the effect of the sudden removal of readers are also presented by analysing the spatial and temporal variation of RFID tracking accuracy. This study reveals the correlation between the testing time and the stability of RFID tracking accuracy. Experimental results show that the proposed approach can be used to assist the RFID system setup process to increase tracking accuracy.

## 1. Introduction

Radio frequency identification (RFID) technology has been identified as cost-effective and has been applied in a number of areas, such as supply chain management and safety tracking [1–4]. However, the insufficient tracking accuracy of RFID is one of the problems impeding wider applications of RFID tracking [5]. Little research has been conducted to analyse the spatial distribution of RFID tracking accuracy, especially regarding the continuous distribution of positioning accuracy. RFID tracking accuracy has often been analysed as a series of discrete unrelated points in a specified area, and the accuracy level between them in the area is left unknown [6,7]. However, a proper and effective analysis on the spatial relationship of these discrete points can describe the continuous spatial distribution of the RFID tracking accuracy. Furthermore, a close examination on the temporal variation of the RFID tracking accuracy can encourage wider applications of RFID.

The aim of the implementation trial in this paper is to identify the spatial and temporal variation of RFID tracking accuracy. To analyse the spatial distribution of RFID tracking accuracy, a test on the RFID tracking accuracy of discrete points in 15 grids was conducted first. Spatial analysis of the relationship among these points was implemented using the Kriging method; as a result, the continuous distribution of the RFID tracking accuracy was presented. Various numbers of readers have been applied during the study at different times to evaluate the variation under different circumstances. The study was conducted on a specified area extending 30 metres wide and 50 metres long at the loading and docking area of a warehouse in Hazelmere, Western Australia.

This paper starts with a background section providing an overview on RFID and its relevant studies on tracking and positioning accuracy (Section 2). This is then followed by an explanation of the study area, equipment and methods used in this study (Section 3). All results and discussion regarding the spatial and temporal variation of RFID tracking accuracy are provided in Section 4. A discussion and conclusions are presented in Section 5, including the significance of the present study and future research required.

## 2. Background

RFID is a potential accurate localisation technology being widely utilised in practice; however, its accuracy needs to be further improved in order to increase the reliability of tracked signals. RFID technology is a state-of-the-art identification approach for various areas, such as supply chain management [8], asset management [9] and ubiquitous computing [10,11], enabled by relatively economical sensing capacities [12]. The basic component of RFID includes readers and tags. The RFID reader, known as an interrogator, is capable of identifying tags individually, then reading or writing digital information into tags. The RFID tag, also called a transponder, is usually a label made with a chip and an antenna. By attaching or mounting tags onto targets to be tracked and retrieving radio frequency waves between the tag and multiple readers, the location of the target can be determined. This would avoid some of the drawbacks associated with other radio frequency (RF) localisation technologies, for example where a direct line of sight is required or where obstacles obstruct the view of a particular reader. A further benefit is that the installation cost is less than other methods [13], such as ultra-wideband (UWB) [14] and wireless local area network (WLAN) [15].

RFID technology itself has been attracting a significant amount of research interest. However, the widespread adoption of RFID for industrial applications is slower than expected, due to its multipath fading issues [16] and the unreliability of the data streams produced by RFID readers [12]. The interrogation signal may result in missing or error information where tags cannot be read or are misinterpreted. Different orientations between tags and reader antennas can also influence the quality of detection. Other than signal transmission, the presence of metal in the vicinity of the tag also limits the use of RFID, since it affects the magnetic flux and further weakens the radio frequency signal [12]. Dobkin and Weigand [17] identify environmental factors, such as metal and liquid around the RFID system, which affect the accuracy of tracked readings. These materials, particularly metal, obstruct the electromagnetic field that the RFID tag and reader rely upon to communicate signals. Water tends to absorb radio waves, making it difficult for tags to collect enough energy to operate. This is especially relevant in the case of its application in liquefied natural gas (LNG) or petrochemical plants, where millions of metal components and liquid containers need to be tracked in their specific locations. These issues show that the identification of RFID accuracy and its error patterns are necessary, in order to make improvements in terms of technical equipment or data processing approaches.

The tracking accuracy of RFID has been identified and improved by a number of previous studies. Zhou and Shi [18] propose a signal propagation model and developed an algorithm based on their study, which maximises the distance accuracy measured between RFID tags and readers. Song *et al.* [19] present an approach using RFID for getting the precise location of a tagged component on construction sites. Depending on the different configuration of tag and tag numbers, the localisation accuracy of RFID varies, but in general, it is 5–9 m with 95% accuracy [20–22]. A variety of errors affect RFID real-time localisation, such as tag collisions, tag detuning, and so forth [11]. Many tag anti-collision approaches have been proposed [23,24]. In order to improve RFID reliabilities, configurable filters have been integrated into RFID middleware to process the data produced by readers [25,26]. All of these efforts have been made to reduce measurement errors and to expand its potential use into more areas that require a stable and high level of accuracy, such as personnel tracking [27] and material tracking [28–31] on the laydown yard of a construction site.

Most of the aforementioned research efforts have been spent on identifying discrete accuracy indices for RFID regarding the entire tracking environment, while few of them focus on spatial and temporal analysis of RFID accuracy distribution. It is crucial to identify where and for how long environment factors influence RFID tracking accuracy. Significant opportunities exist to discover spatial and temporal patterns of RFID tracking accuracy, in an attempt to further improve positioning quality. This motivated the research presented in this paper, and an implementation trial with an active tags RFID system has been conducted for data analysis.

## 3. Methodology

### 3.1. Study Area

**Figure 1.**
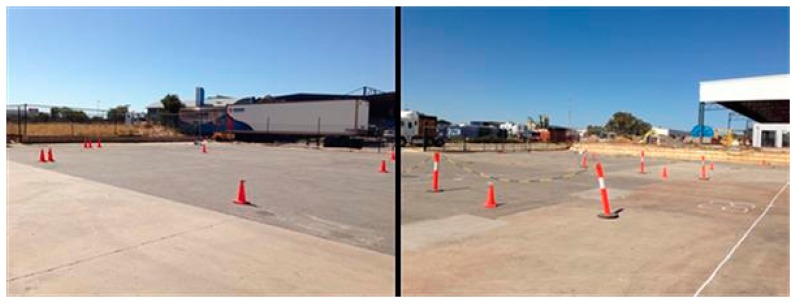
Field view of the study area.

**Figure 2.**
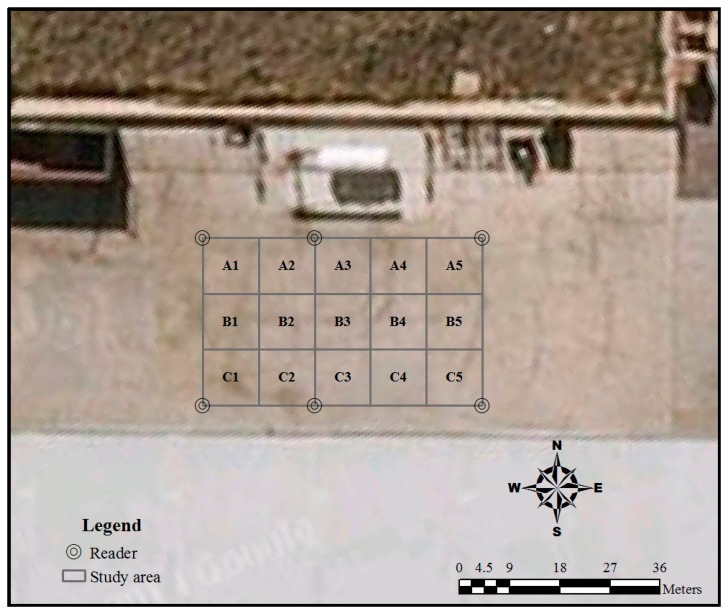
Sub-divided study areas with their codes.

A study area (50 m long and 30 m wide) was chosen to evaluate the spatial and temporal variation of the RFID tracking accuracy. The study area was located in front of a warehouse in Hazelmere, Western Australia, Australia (Figure 1). The area was divided into twelve 10 × 10-m grids, and each grid was coded, as Figure 2 shows.

### 3.2. Equipment and Outline of Methods

**Figure 3.**
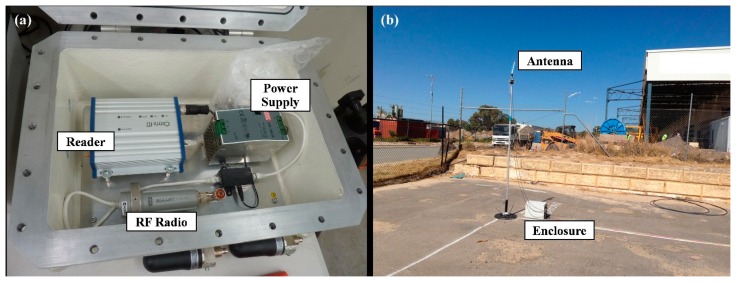
Hardware components: (**a**) the layout of the enclosure; (**b**) antenna and enclosure.

**Figure 4.**
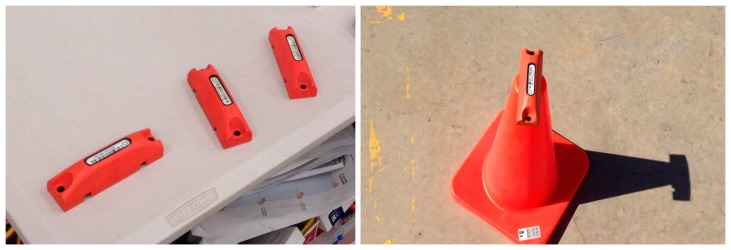
Example of the RFID tags used in this study.

The design of the testing process includes the following hardware components:

- RFID reader (as shown in Figure 3a);
- 2.6 metre-high 433 MHz Antenna (as shown in Figure 3b);
- Active RFID tag (as shown in Table 1 and Figure 4);
- RF radio (operating at 2.4 GHz, for Wi-Fi communications);
- 2.4 GHz antenna (for Wi-Fi communications, as shown in Figure 3b);
- Power management, including:
  - 240-V AC to 24-V DC Mean Well power supply;
  - Power Over Ethernet (PoE) injector;
- 240-V AC power cable and cabling from the components to the antennas.

The reader, RF radio and devices for power management are all contained in an enclosure. The enclosure is connected with antennas, which are mounted on a pole, as shown in Figure 3. The RFID tag is a dual band tag that can be used in either active or passive mode. In this study, it is used as an active tag. The tag will emit a signal, and the position of the tag can be identified by receiving this signal with multiple readers in the study area. The tag is designed specifically for an oil and gas environment. As can be seen in Figure 4, the rugged tag is coloured for high visibility and tested up to 4000 tons of pressure. It can be operated between −40 to 60 degrees Celsius. The tag is ideal for applications requiring long-range asset tracking, particularly in harsh environments, such as LNG plants.

An RFID reader was placed at each of the four corners of the study area, and two more readers were placed on the top right corner of the A2 grid and the right bottom corner of the C2 grid to test the variation of RFID accuracy with different numbers of readers. The workflow of the methods applied in this study is outlined as follows:

1. Set up the equipment and divide the study area into 15 sub-areas;
2. Conduct a test on the RFID positioning errors in each sub-area by field work;
3. Analyse the spatial distribution of RFID tracking accuracy;
4. Evaluate the spatial and temporal variation of RFID tracking accuracy.

### 3.3. Evaluation of RFID Accuracy by Field Work Design

The test process started with 4 readers. Fifteen tags were randomly placed in different grids of the study area. The error of the RFID was measured by the difference between its actual position and the position read on the RFID system. The position information shown on the RFID was recorded more than once with different time intervals to evaluate the temporal variation of the RFID accuracy. The same process was conducted again with a different number of readers to assess the robustness of the testing results.

However, as illustrated in Section 2, the tracking accuracy of RFID in all of the previous studies was collected in the form of discrete data. The accuracy information nearby the observed points is left unknown. The previous study shows that compared with discrete representation, the continuous representation of the spatial and temporal variation can reveal more information [32]. Therefore, to investigate the performance of RFID, a continuous representation of the spatial variation of the RFID accuracy is required.

The Kriging method was used to interpolate data gaps in ground profiles. Compared to other interpolation methods, such as nearest neighbour, spatial averaging and inverse distance weighting, Kriging [33,34] has been proven to provide more accurate (minimum variance) and less biased estimates when applied to beach morphology [35,36]. However, Kriging may rely on the smoothness assumption of the interpolated surface [37], and it is restricted to modelling the first and second order effects in spatial analysis [38]. Nonetheless, Li and Heap [37] state that when observations are insufficient to compute variograms, the gap in sparse data can be satisfactorily interpolated using the Kriging method.

### 3.4. Kriging Method

The first step of the evaluation method on spatial and temporal variation of RFID accuracy is to interpolate a number of data points using the Kriging method. Kriging is used to predict the missing RFID accuracy information by the sum of the surrounding weighted values of the observed tag position data [35]:

(1) $$\hat{Z}(s_{0})=\overset{N}{\underset{i=1}{\text{∑}}}\lambda_{i}Z(s_{i})$$

where *Z*(*s_i_*) is the observed value at location *i*, *λ_i_* is the weight at the location *i*, and the sum of *λ_i_* is 1, therefore the optimal values of *λ_i_* can be calculated by the method of Lagrange multipliers. *s*_0_ is the location to be estimated and *N* is the number of observed data used to predict *Ẑ*(*s*_0_).

The weights *λ_i_* were estimated based on spatial autocorrelation theory using semivariogram models as follows [35,39]:

(2) $$\gamma \left(h\right)=\frac{1}{2}\text{Var}\left\{F\left(x_{i}\right)-F\left(x_{i+h}\right)\right\}$$

where γ(*h*) is the estimated variance between two observed data points, *h* is the distance between two observed data points and *F*(*x*) represents the semivariogram function. In this study, the centre of each grid is considered as location *i*, where the observed value is. All of the other areas are considered as the location to be estimated.

Modelling the continuous semivariogram function is the key step between the spatial description and spatial prediction [40]. The basic principle of spatial autocorrelation in this research means the tracking accuracies at two locations that are closer to each other are more alike than those further apart. Therefore, as pairs of locations become further apart, the squared difference of the tracking accuracy values at these two locations will increase correspondingly. This presents the correlation between semivariogram values and the distances between two locations. As the empirical semivariogram does not provide information for all of the distances, a continuous function is required to fit the empirical semivariogram.

A number of semivariogram functions are available to fit the empirical semivariogram, including circular, spherical, exponential, Gaussian, linear, *etc*. Each model is designed to explain different types of phenomena. The most suitable model should reveal the general correlation between the variation of the observed information (such as the tracking accuracy of RFID) and the variation of distance. Valeriano *et al.* [41] estimated that the Kriging method with a Gaussian semivariogram model can produce a smoother interpolated surface than the other semivariogram functions. As the RFID tracking accuracy is supposed to be smoothly distributed in the testing area, the Gaussian function was applied in this study to fit the empirical semivariogram.

## 4. Data Analysis and Discussion

### 4.1. Field Work Test on RFID Accuracy

**Figure 5.**
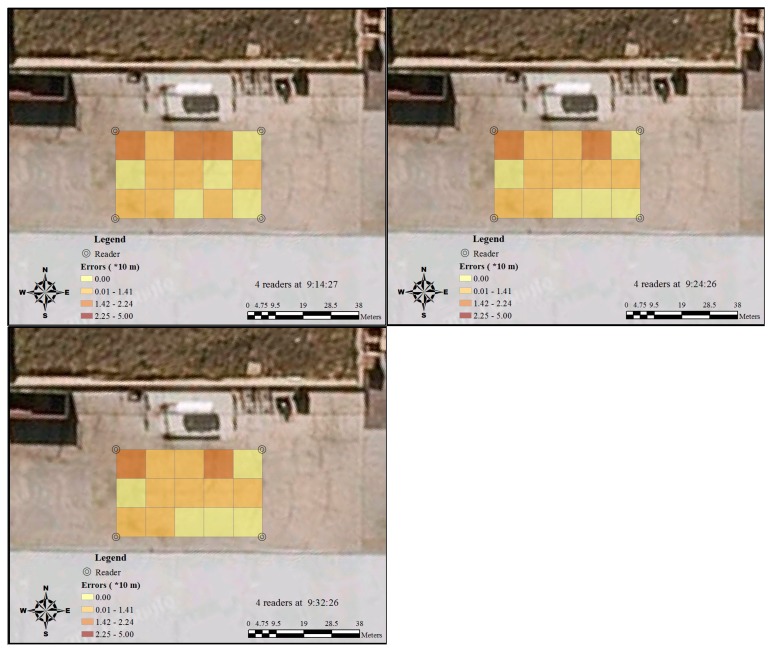
Temporal variation of RFID errors distribution in the discrete grids by four readers.

**Figure 6.**
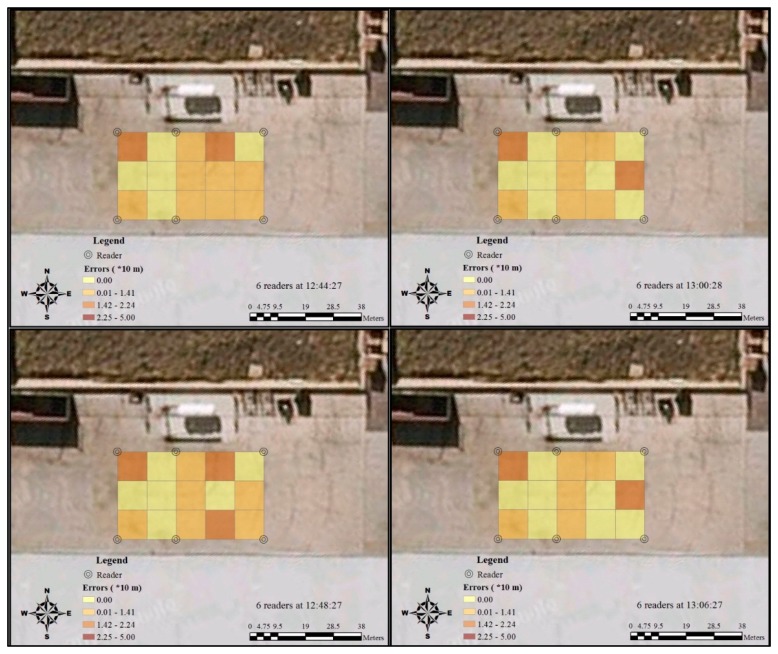
Temporal variation of RFID error distribution in the discrete grids by six readers.

**Figure 7.**
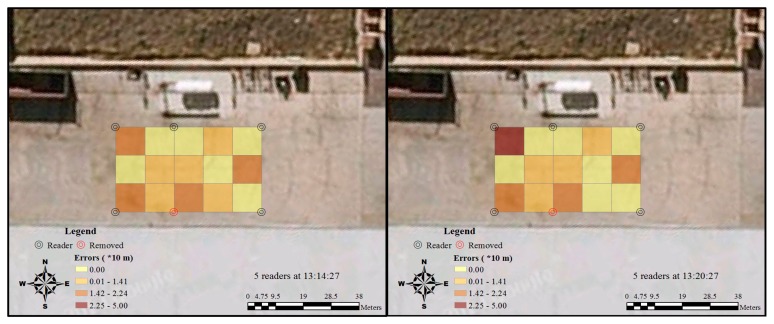
Temporal variation of RFID errors distribution in the discrete grids by five readers.

The testing was conducted with 15 RFID tags on 4 March 2014. Tables 2 and 3 show the actual positions of tags and their positions detected by the RFID system, using 4, 6 and 5 readers, respectively. The information provided in these two tables delivers the most initial records of the RFID tracking accuracy. Figures 5, 6 and 7 provide more visualised information about the accuracy results that are more easily interpreted. Fifteen RFID tags were placed in different grids, and their positions detected by readers were shown on the RFID system. The distances were measured between the centre of the grids where the tag is and the RFID system detects. They were classified into four groups, ranging from 0 to 50 m. As Tag 1212 was not detected by five readers at 13:20:27, the largest potential error, 44.72 m, was applied in this case.

In Figure 5, more areas were identified as catalogue three (14.2 m–22.4 m) when the RFID system was first set up with four readers. As time passed, the number of this catalogue decreased, and the accuracy pattern became more stable. This phenomenon was confirmed by another test with six readers (Figure 6). The additional two readers significantly increased the accuracy of RFID positioning in Grids A2, B2, C2. After the reader at the right bottom of C2 was removed, the positioning accuracy in the surrounding area declined correspondingly (Figure 7). During the whole testing period, the accuracy at Grid A1 was consistently unsatisfying.

The previous testing shows the general distribution of the RFID accuracy in the study area, while fine resolution can provide more information about the spatial variation of the RFID and identify the area that needs to be augmented in terms of the RFID signal.

### 4.2. Spatial and Temporal Variation of RFID

**Figure 8.**
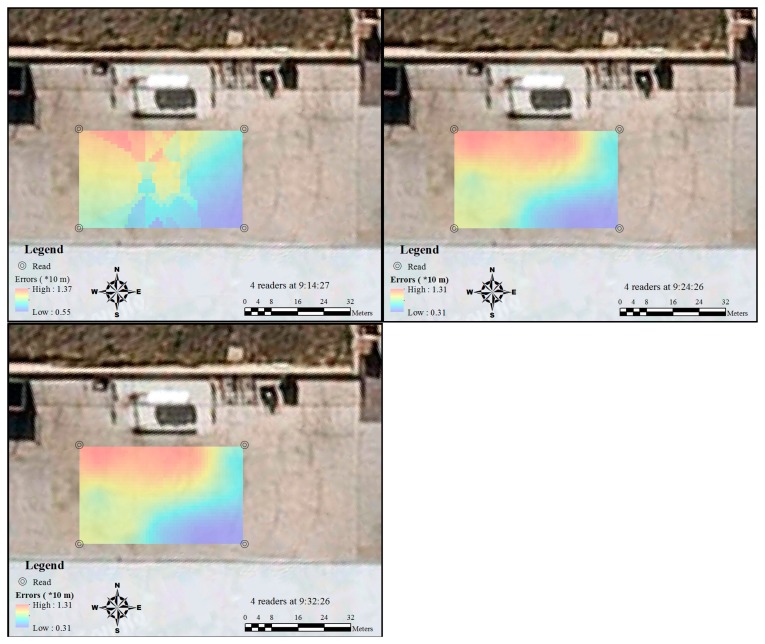
Spatial and temporal variation of RFID errors distribution by four readers.

The Kriging method was applied in this study to interpolate the unknown accuracy value in the study area with the 15 recorded values in the regular grids. The interpolated accuracy field with fine resolution in the study area provides more information that can be interpreted. The results identify the clusters of high and low RFID tracking accuracy under different situations in the testing area. Figure 8 shows that 10 min after the first record of the test results, the error distribution of the RFID became stable and the interface between high and low accuracy areas became smoother. Furthermore, the overall accuracy increased in terms of both the lowest and highest values of RFID tracking accuracy.

**Figure 9.**
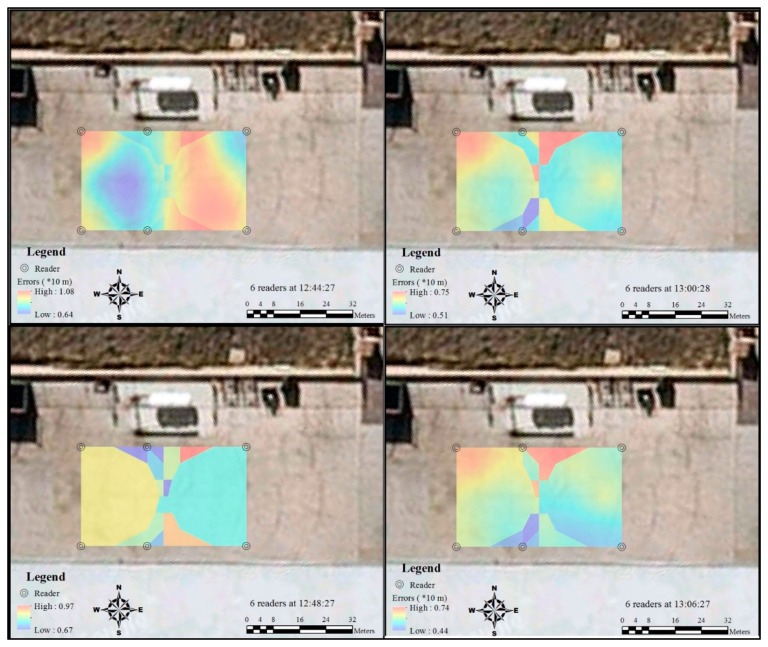
Spatial and temporal variation of RFID errors distribution by six readers.

When another two more readers were placed in the middle of the study area (closer to the A1 and C1 sides), the smooth accuracy distribution was interrupted (Figure 9). The accuracy around the two new readers and the area close to the middle of Grids B1 and B2 significantly increased. This phenomenon lasted the whole testing period with the six readers. In contrast, the accuracy level in the area around Grids B4 and B5 changed dramatically during the testing period. When the two extra readers were first placed, the RFID error was still at a high level, and this level only dropped 4 min after the first test, when the accuracy in this area became stable; however, the overall accuracy in this area was still lower than the other side, which was close to the middle of Grids B1 and B2. This can be partly attributed to the difference in the distances between readers. On one side, the gap distance for the better results is 20 m, while the other is 30 m. During the whole testing period, both the largest error and the lowest error declined, and the largest errors that occurred were smaller than the situation when only four readers were used.

**Figure 10.**
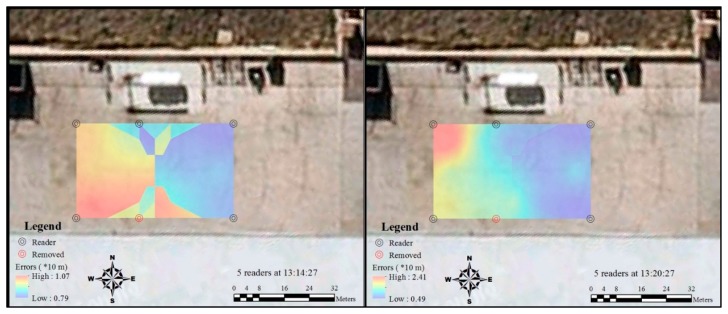
Spatial and temporal variation of RFID errors distribution by five readers.

After the reader at the bottom of C2 was removed, the error suddenly increased in terms of both largest and lowest values (Figure 10). The removal of the reader had an impact in the area around it. However, the lowest accuracy at this moment was even worse than the period with four readers. The lowest accuracy level got better 6 min after the removal of the reader. As the position of the tag in A1 was not detected in the second test with five readers, the large assumed value at A1 may have an impact on the largest accuracy value in this test. However, it still can be identified that the interface between different accuracy levels became smoother when the test time was of a longer duration. In addition, compared with the grids of the B1 and B2 side, the removal of the reader had less impact on the grids of the B4 and B5 side.

Table 4 shows the overall test results with different numbers of readers and at different times. These results are calculated based on Kriging interpolation of the RFID accuracy at the study area. The accuracy levels listed in this table indicate the average RFID errors in the whole testing area. It can be estimated that the RFID accuracy with six readers is much better than the others. Regarding the test with four and six readers, the accuracy became higher as the testing time was increased. When one of the readers was removed and only five readers were left, the accuracy was even worse than the situation when only four readers were set up. However, whether the accuracy level could become more stable and higher requires further study with a longer test period. In Table 5, the root mean square error (RMSE) was employed to validate the results presented. It can be identified that with the same number of readers, the interpolation results get better as the testing time increases. On the other hand, both the removal and addition of readers will have a negative impact on the Kriging interpolation of the RFID accuracy.

During the whole period of testing, the results in the area around Grid A1 were consistently unsatisfying. The reason can be attributed to the presence of metal materials in the vicinity. It is also possible that other radio frequency sources could partially interfere with the RFID signals in the study area. The results of this study show that identifying the continuous distribution of the RFID accuracy is necessary, given the circumstance that spatial variations of the RFID accuracy may exist. The continuous distribution of the RFID tracking accuracy provides more information than the discrete accuracy test.

### 4.3. Potential Applications for Spatial and Temporal Analyses of RFID

The spatial and temporal analysis of RIFD positioning accuracy demonstrates the ability to discover potential error sources, such as environmental factors, in the study area. It can be applied to RFID system design and used to assist in the decision-making process, thus increasing positioning accuracy in harsh environments. There are further applications for this research in construction management, where tracking construction material, vehicles, equipment and personnel can also benefit from this study. The interference factors affecting RFID accuracy on construction sites can be identified and avoided when setting up the RFID system. Integrations of accurate RFID tracking systems and other enabling technologies can assist with improving the productivity of current construction, especially on large-scale and complicated sites, such as LNG plant constructions [31]. For example, accurate RFID tracking systems can be utilised to identify and detect the materials in laydown yards, even where there is a mismatch between information from the work package and where the material is actually located. This can be enhanced by combining location information with handheld devices and mixed reality (MR) technologies [42–44]. In this situation, work crews can efficiently and effectively locate the required materials with the assistance of visualised interfaces [45–47]. The RFID tag on/off records from the construction crews can show the accurate status of a construction site, by checking whether the work tasks are being performed appropriately or in the right place and right time. In addition, worker safety issues can be addressed when the duration of working time for each work crew can be accurately monitored by the RFID tracking system. In this system, warning messages will be sent to construction managers or crews to avoid the risk of overwork, which will finally improve the overall productivity of the construction.

## 5. Conclusions

This study quantitatively assesses the spatial and temporal variation of the RFID accuracy with different numbers of readers. Spatial analysis was applied in this research to interpolate the unknown accuracy level in the study area. The results identified in this study reveal the continuous distribution of the RFID accuracy level within the RFID testing area. This provides more information about the location of areas with weak signal covers, which can be improved to increase the overall RFID accuracy. Other significant conclusions include: (1) 20 m has been identified in this research as an ideal gap distance between two readers; (2) the sudden removal of a reader has a significant impact on the overall accuracy; (3) the longer the testing time lasts, the higher and more stable the RFID accuracy. The potential applications of the precise identification of RFID tracking were also presented. Regarding the temporal analysis in this study, a longer time span and more records in this time span are required to confirm the conclusions made in this research.

## Figures and Tables

**Table 1.** The specifications of the RFID tag used in this study.

**Radio**	**Active**
**Power**	5 years or 5 million transmits under normal use
**Frequency Range**	433 MHz
**Read Range**	Up to 400 m
**Memory**	n/a
**RFID**	Proprietary

**Table 2.** Comparisons of the tags' actual position and located by RFID using four readers at different times.

**Tag #**	**Actual Position**	**March 4 9:14:27**	**March 4 9:24:26**	**March 4 9:32:26**
**1205**	B2	C2	C2	C2
**1206**	B5	B4	B4	B4
**1212**	A1	C1	C1	C1
**1214**	B1	B1	B1	B1
**1396**	C1	B1	B1	B1
**1398**	A2	B2	B2	B2
**1399**	C2	B3	B3	B3
**1466**	A4	C4	C4	C4
**1468**	A5	A5	A5	A5
**1644**	C5	C5	C5	C5
**1201**	C4	C5	C4	C4
**1442**	B3	B2	B2	B2
**1557**	B4	B4	B5	B5
**1591**	A3	B5	B4	B4
**1672**	C3	C3	C3	C3

**Table 3.** Comparisons of the tags' actual position and location by RFID using six and five readers at different times.

**Tag #**	**Actual Position**	**March 4 12:44:27**	**March 4 12:48:27**	**Restart all Readers March 4 13:00:28**	**March 4 13:06:27**	**5 Readers, C2 Reader off March 4 13:14:27**	**5 Readers, C2 Reader off March 4 13:20:27**
**1205**	B2	B2	B2	B2	B2	A2	A2
**1206**	B5	B4	B4	B3	B3	B3	B3
**1212**	A1	C1	C1	C1	C1	C1	Not in grid
**1214**	B1	B1	B1	B1	B1	B1	B1
**1396**	C1	B2	B2	B1	B1	A1	A1
**1398**	A2	A2	A2	A2	A2	A2	A2
**1399**	C2	C2	C2	C2	C2	B3	B3
**1466**	A4	A2	A2	A3	B3	B3	A3
**1468**	A5	A5	A5	A5	A5	A5	A5
**1644**	C5	B4	B4	C5	C5	C5	C5
**1201**	C4	B3	B2	B4	C4	B4	C4
**1442**	B3	B2	B2	B2	B2	A2	A2
**1557**	B4	A4	B4	B4	B4	B4	B4
**1591**	A3	A2	A2	A2	A2	A3	A3
**1672**	C3	C2	C2	C2	C2	A2	A2

**Table 4.** The overall test results with different numbers of readers and at different times.

	**4 Readers Test 1**	**4 Readers Test 2**	**4 Readers Test 3**	**6 Readers Test1**	**6 Readers Test 2**	**6 Readers Test 3**	**6 Readers Test 4**	**5 Readers Test 1**	**5 Readers Test 2**
**Accuracy (m)**	9.31	8.62	8.62	8.69	8.44	6.4	5.95	9.25	10.23

**Table 5.** RMSE of Kriging interpolation on RFID accuracy.

	**4 Readers Test 1**	**4 Readers Test 2**	**4 Readers Test 3**	**6 Readers Test1**	**6 Readers Test 2**	**6 Readers Test 3**	**6 Readers Test 4**	**5 Readers Test 1**	**5 Readers Test 2**
**RMSE (m)**	6.84	6.15	4.69	7.25	9.25	6.74	7.67	8.91	13.83

## Disclaimer

The views expressed in this paper are the authors' personal views and are not attributable to Woodside. The paper is intended to be a general discussion and does not constitute advice. Information has not been verified by Woodside. No representation or warranty is made as to the accuracy, completeness or reliability of the information. All liability for any loss or damage incurred by any person as a result of using, acting on, relying on or disclosing any of the information contained in this paper or otherwise in connection with this paper is disclaimed.
